# Identification of TGFBR1 Gene Variants in Two Chinese Pedigrees with Loeys-Dietz Syndrome

**DOI:** 10.21470/1678-9741-2023-0495

**Published:** 2025-02-05

**Authors:** Jiehua Qiu, Wei Chen, Xixi Min, Yang Shen, Xianhua Zhu, Jiacong Qiu, Xiande Zeng, Xiong Zeng, Yanchun Ji, Weimin Zhou

**Affiliations:** 1 Department of Vascular Surgery, the Second Affiliated Hospital of Nanchang University, Nanchang, Jiangxi, People’s Republic of China; 2 Medical College, Nanchang University, Nanchang, Jiangxi, People’s Republic of China; 3 Department of Genetic Medicine, the Second Affiliated Hospital of Nanchang University, Nanchang, Jiangxi, People’s Republic of China; 4 Jiangxi Key Laboratory of Molecular Medicine, the Second Affiliated Hospital of Nanchang University, Nanchang, Jiangxi, People’s Republic of China; 5 Division of Medical Genetics and Genomics, The Children’s Hospital, School of Medicine, Zhejiang University, National Clinical Research Center for Child Health, Hangzhou, People’s Republic of China

**Keywords:** Loeys-Dietz Syndrome, Transforming Growth Factor-beta Type 1, Lysine, Computed Tomography Angiography, Exons, Genetic Testing, High-Throughput Nucleotide Sequencing, DNA

## Abstract

**Objective:**

To investigate a precise treatment and related gene variants in some Loeys-Dietz syndrome (LDS) patients with vascular disease.

**Methods:**

Two probands (JX001-II1 and JX002-II1) diagnosed with LDS and their families were recruited. Routine blood test, antiphospholipid antibodies, immune globulins, nuclear antibodies (ANAs) and biochemical tests, and computed tomography angiography (CTA) were performed for probands. Deoxyribonucleic acid was collected from the two families and was sequenced by the next-generation sequencing employing the Ion Torrent platform (Life Technologies); the variants were confirmed by Sanger sequencing.

**Results:**

Two probands’ antiphospholipid antibodies, immune globulins, and ANAs were near normal. CTA showed that both probands had an LDS patient typical arterial change: aortic aneurysm. Genetic testing of the 10 LDS-associated genes in the two probands showed that c.605C>T (JX001-II1) and c.679G>A (JX002-II1) variants were both positioned in exon 1 of TGFBR1 and it results in the substitution of highly conserved 202 alanine (Ala) for valine (Val) ( P. Ala 202Val, JX001-II1) and 227 glutamic acids (Glu) for lysine (Lys) ( P. Glu 227Lys, JX002-II1). However, the parents of both patients did not have similar symptoms and did not carry such gene variants. Proband 1 (JX001-II1) died unexpectedly during the operation preparations, whereas proband 2 (JX002-II1) underwent two operations, and the patient is currently in excellent health.

**Conclusion:**

The two TGFBR1 gene variants may be a primary genetic cause of LDS. The results expand the TGFBR1 variant spectrum. Endovascular surgery can be a feasible option for LDS patients.

## INTRODUCTION

Loeys-Dietz syndrome (LDS), an autosomal dominant genetic disorder, is linked with variants in a signaling pathway termed transforming growth factor-β (TGF-β) involving variants in TGF-β receptors, TGFBR1 & TGFBR2, TGF-β ligands, TGFB2 & TGFB3, and SMAD2 and SMAD3^[1,2,3,4]^. The LDS, caused by the variants in TGFBR1 and TGFBF2, was initially identified as type I and type II, respectively^[5]^. Patients with type I LDS had craniofacial anomalies, for instance, hypertelorism and a broad, bifid uvula or cleft palate, along with vascular disorders, *viz.*, vascular aneurysms, dissection, and tortuosity^[6,7]^. LDS patients are at risk of acute aortic events^[8]^, *e.g.*, the aortic rupture carries a substantial risk of premature mortality^[5]^. TGF-β, a family of multipotential cytokines, controls diverse cellular destinies, *viz.*, cell death, repertoire, migration, and proliferation^[1,2]^. The binding of TGFBR1 and TGFBR2 to TGF-β ligands activates signaling pathways, which are transmembrane threonine and serine kinases^[9]^ and are composed of a transmembrane region and extracellular (cysteine-rich) and intracellular (kinase) domains. To date, very few cases (around ten^[5,10]^) with TGFBR1 variants have been reported, despite the number of studies that described solely LDS patients without specifying the exact genetic cause. The current study reports two young adult female LDS patients having aortic aneurysm and dissection, as well as the identification of two TGFBR1 gene variants.

## METHODS

### Subjects, Laboratory, and Imaging Examinations

**Fig. 1:**
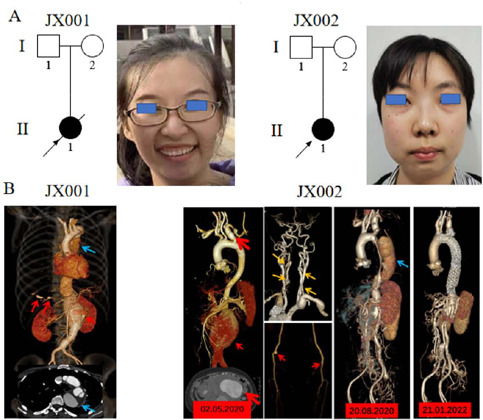
A) Samples from the patients belonging to two Han Chinese families (JX001 & JX002) and their facial features. B) Computed tomography angiography test showed the following disorders in two patients: Standford Type B aortic dissection (blue arrows), thoracoabdominal aortic aneurysm (red arrows in Figure 1B, right), hepatic arterial aneurysms (red arrows in Figure 1B, left), and tortuous vertebral arteries (yellow arrows).

Figure 1 depicts that samples from the patients belonging to two Han Chinese families (JX001 & JX002) were collected by the Department of Vascular Surgery, at the Second Affiliated Hospital of Nanchang University. Control deoxyribonucleic acid (DNA) samples (100) were also collected from a group of unaffected subjects belonging to the Han Chinese ancestry of the same region. Several tests were conducted on the samples collected from these families, which include a routine blood test, biochemical test, test for antiphospholipid antibodies, immune globulins, antinuclear antibodies (ANAs), antineutrophil cytoplasmic autoantibodies (ANCAs), and rheumatoid factor (RF), along with electrocardiography, computed tomography angiography (CTA), and clinical evaluations of probands^[11,12,13]^. In addition to sample collections and clinical evaluations, informed consent was obtained from these patients, and all protocols established by the Second Affiliated Hospital of Nanchang University's Ethics Committees (approval number: 20230623) were followed.

### DNA Sequencing and Variants Identification

**Fig. 2:**
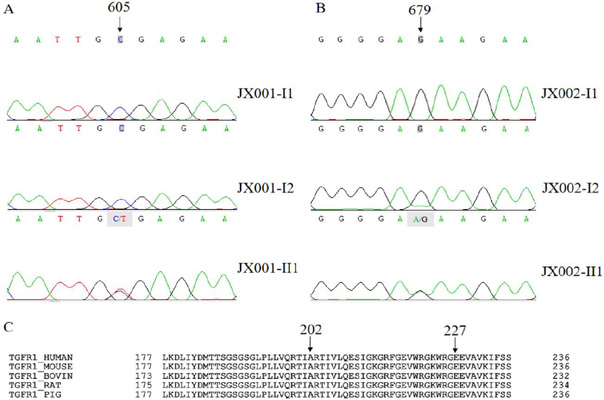
Different gene mutation sites in JX001-II1 and JX002-II1.

**Fig. 3:**
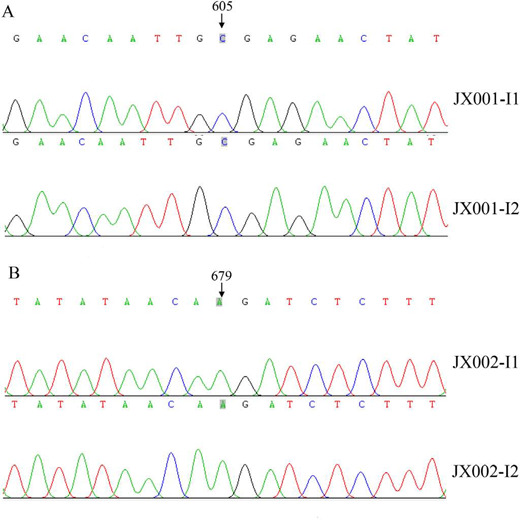
Partial sequence chromatograms of TGFBR1 gene from the proband’s parents in pedigree JX001 and pedigree JX002. An arrow indicates the location at position 605 and 679 in TGFBR1 gene.

DNA was sequenced by the next-generation sequencing (NGS) employing the Ion Torrent platform (Life Technologies) (Figure 2, Figure 3), and the variants were confirmed by Sanger sequencing. A blood DNA extraction kit (TIANGEN, Beijing, China) was employed for DNA extraction from peripheral blood samples of 200 µL.

According to the standard protocol, for the preparation of the library with the Ion AmpliSeqTM Library Kit 2.0 (Thermo Fisher Scientific, United States of America), 10 ng DNA was used for every sample, and the same amount was used for customized familial thoracic aortic aneurysm and dissection-related gene panel. This panel contains amplicons, covering the coding regions and intron/exon boundaries in 10 genes (TGFB3, TGFB2, PRKG1, TGFBR2, TGFBR1, SMAD3, MYLK, MYH11, FBN1, and ACTA2)^[14,15,16,17,18,19]^. Ion personal genome machine system, equipped with an ion 318 chip with at least 200× sequence coverage, was used for sequencing.

Torrent Suite Software (Thermo Fisher Scientific, United States of America) was utilized for variant calling and genome alignment (*i.e.*, mapping to the reference genome hg19).

After ward, variant filtering and annotation were performed through Ion Reporter Suite (Thermo Fisher Scientific, United States of America). All detected uncommon variants with an allele frequency of < 1% were evaluated and interpreted following the variant categorization recommendations of the American College of Medical Genetics and Genomics (ACMG)^[20]^. NGS detected putative pathogenic variants which were validated employing Sanger sequencing using a 3500 Genetic Analyzer (Applied Biosystems, United States of America), based on the standard procedure. Specific primers, having sequences 5'-CACTCATTAGTGCCTATCATGATGTT-3' (for forward primer) and 5'-AGGAATGCTATCAAGAGTCAAGAAAA -3' (for reverse primer), were designed to amplify fragments, including variants in TGFBR1 (NM_004612.4).

### Phylogenetic Analysis

The interspecific analysis of the TGFBR1 gene sequences of five distinct vertebrates using UniProt (https://www.uniprot.org/) was conducted according to the reported technique^[21,22]^.

With the comparison of human nucleotide variants with those of the four other vertebrates, conservation index was calculated, which was then known as the percentage of the species from the list of four different vertebrates having conserved wild-type nucleotide position^[23]^.

## RESULTS

### Clinical Presentation

**Proband 1** (JX001-II1), a 36-year-old female, was admitted to the hospital due to laceration pain in the chest and back. C TA test showed the following disorders: Standford Type B aortic dissection, thoracoabdominal aortic aneurysm (TAAA), hepatic arterial aneurysms, and tortuous vertebral arteries (Figure 1B). Results of laboratory examinations showed that hemoglobin was 72 g/L, and levels of antiphospholipid antibodies, immunoglobulins, ANCAs, ANAs, and RFs were also normal. In addition, the findings of additional laboratory testing were normal. Neither of the patient's parents suffered from a vascular illness.

**Proband 2** (JX002-II1), a 31-year-old female, was admitted to the hospital due to abdominal pain. The CTA revealed the presence of the following conditions: TAAA, popliteal artery aneurysm, and left subclavian artery aneurysm, combined with tortuous vertebral arteries. Furthermore, TAAA was ruptured (Figure 1B). The laboratory exams revealed that the patient's hemoglobin level was 72 g/L, and like the other laboratory examinations, levels of antiphospholipid antibodies, immunoglobulins, ANCAs, ANAs, and RF were normal. She was given first operation treatment and presented intracranial bleeding in the perioperative time. The patient experienced sudden back pain three months after surgery, and additional C TA scans revealed a Standford Type B dissection (Figure 1B). Based on these results, endovascular therapy was administered to the patient. The patient's parents are living an everyday healthy life without any vascular disorder.

### Identification of TGFBR1 Variant

Targeted gene sequencing (including TGFB3, TGFB2, PRKG1, TGFBR2, TGFBR1, SMAD3, MYLK, MYH11, FBN1, and ACTA2) of the probands JX001-II1 and JX002-II1 was performed to identify the genetic cause. The identified rare missense variants c.605C>T (JX001-II1) and c.679G>A (JX002-II1) are both positioned in exon 1 of TGFBR1 (NM_004612.4) and it results in the substitution of highly conserved 202 alanine (Ala) for valine (Val) (P.Ala202Val, JX001-II1) and 227 glutamic acids (Glu) for lysine (Lys) (P.Glu227Lys, JX002-II1) (Figure 2). Sanger sequence analysis of DNA fragments covering all exons along with the flanking sequences of TGFBR1 established that symptomatic subjects carried the heterozygous c.605C>T (JX001-II1) and c.679G>A (JX002-II1) variants. According to the ACMG guidelines for the interpretation of sequence variants^[23]^, P.A202V and P.E227K were assessed to be likely pathogenic and of uncertain significance (suspected pathogenic), respectively. However, neither of their parents (JX001-I1, JX001-I2, JX002-I1, or JX002-I2) carried the TGFBR1 variant.

### Treatment and Prognosis

Proband 1 (JX001-II1) died unexpectedly during the operation preparations, whereas proband 2 (JX002-II1) underwent an open operation for the treatment of TAAA, which consisted of a bypass from thoracic aorta to both iliac arteries, both renal arteries, and a superior mesenteric artery. During the perioperative and therapeutic phases, the patient (proband 2) experienced minor cerebral hemorrhage. The patient recovered and was discharged after one month. Three months later, the patient underwent thoracic endovascular aortic repair (TEVAR) (Gore graft excluders, W. L. Gore & Associates, Flagstaff, Arizona, United States of America) and embolism of a subclavian artery for the novel Standford type B aortic dissection (Figure 1B). Following the second operation, the patient's recovery was excellent. Subsequently, the patient received follow-up care every three to six months, and she is currently in excellent health.

## DISCUSSION

The current study describes two Chinese women who have been diagnosed with LDS. We described two unique single-nucleotide polymorphism (SNP) variants of TGFBR1 in two young female patients with type I LDS, validated by genetic testing and clinical features. Two novel heterozygous missense pathogenic variants on c.605C>T (P.202Ala>Val) and c.679G>A (P.227Glu>Lys) of TGFBR1 were identified through genetic studies, as similar with two Italian cases and a Korean family, respectively^[24,25]^. Initially, *in vitro* evaluations of the pathogenicity of these variants were conducted, noting that the patient had a c.1613T>C(Val538Ala) variant of TGFBR2 with LDS. Moreover, both the patients were admitted with TAAA and Standford Type B aortic dissection and other artery aneurysms as emergency conditions. One patient failed to get treatment, while the other got the treatments for the aortic disease, leading to better recovery until now.

**Fig. 4:**
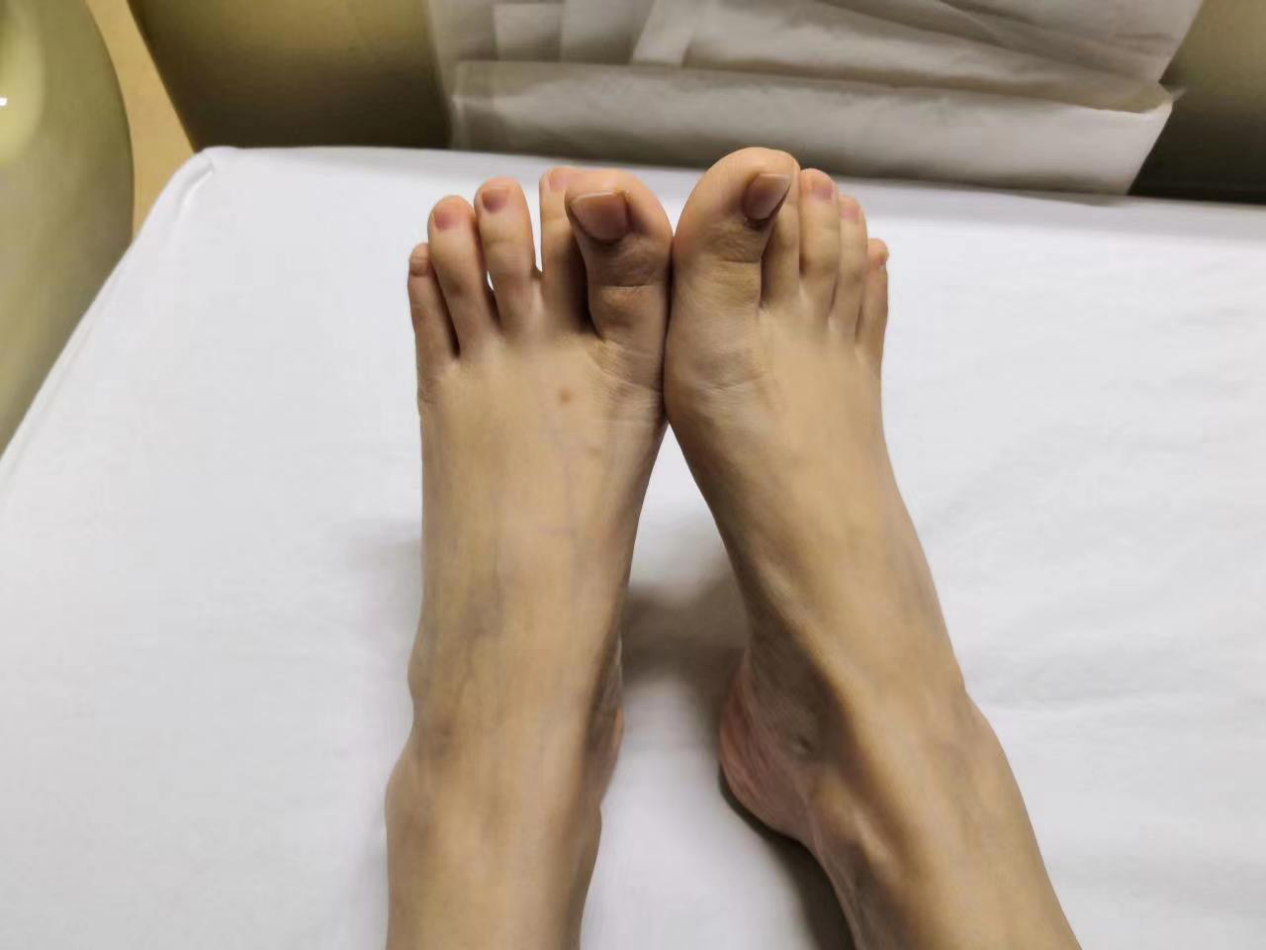
Proband JX001-II1’s skeletal symptoms (long soles and toes).

**Fig. 5:**
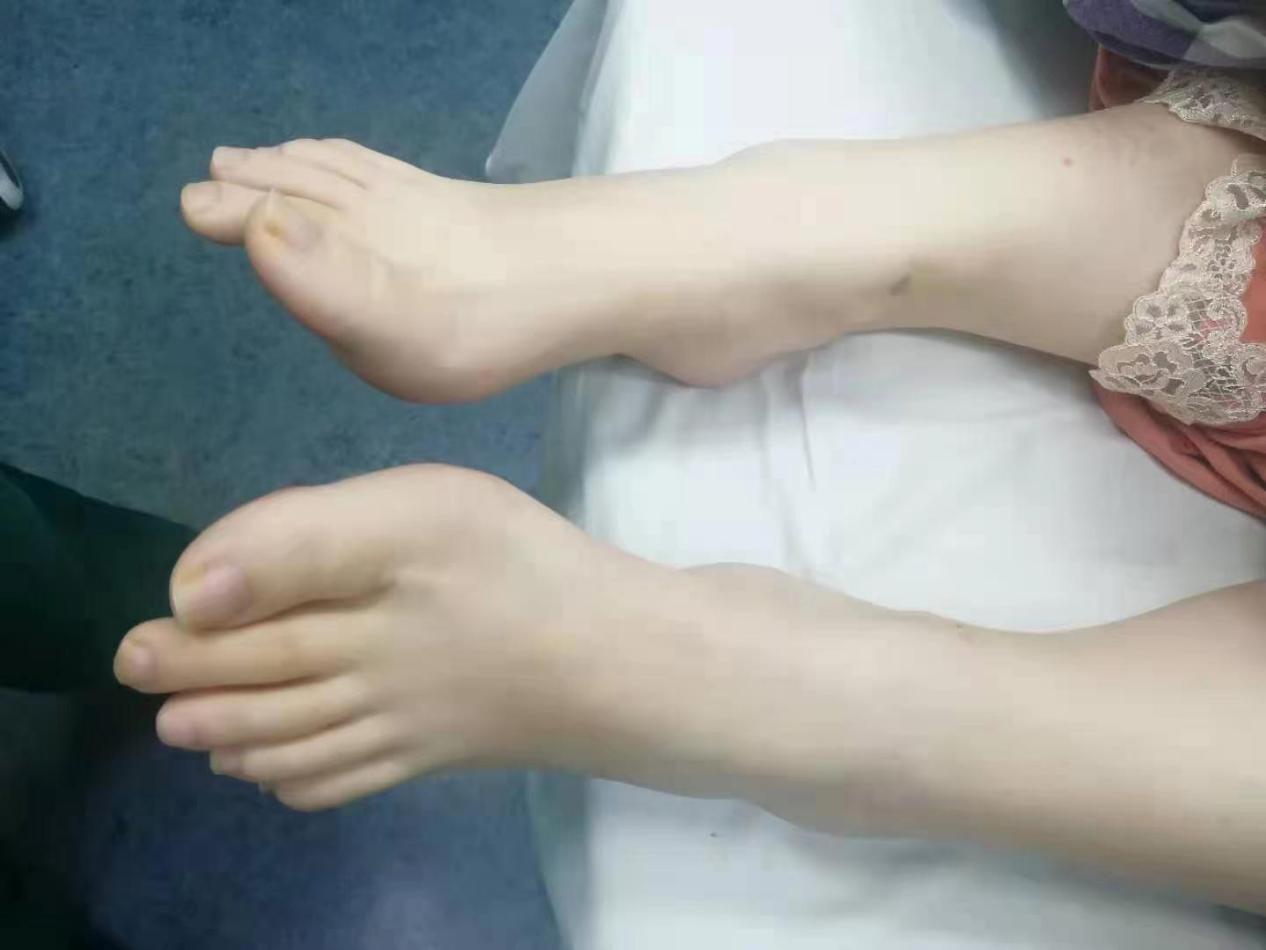
Proband JX002-II1’s skeletal symptoms (long toes and deformed soles).

LDS implies autosomal dominance with various clinical manifestations. LDS patients are at high risk of various disorders, for instance, aortic aneurysm, dissection, or rupture at an early age. In addition, they may have widely spaced eyes (Figure 1A), a cleft palate, a bifid uvula, generalized arterial tortuosity, mental retardation, aneurysms throughout the arterial tree^[5,7,10,11,12]^, and thin and long toes (Figure 4, Figure 5). It was reported that Standford Type B dissection was the first aortic event in 1.7% of type I LDS population^[12]^, whereas only 10% of LDS patients have been diagnosed with aortic aneurysms^[5,11]^. About 92% of LDS patients also had aneurysms of other arteries^[11]^. According to the study of Jeffrey A Jones et al.^[26]^, genetic mutations of TGF-β leads to dysregulation in the processes that maintain vascular integrity, which potentiates extracellular matrix degradation and increases susceptibility to aortic dilatation and dissection as well as the other clinical features. It well explained the susceptibility of LDS patients to aortic disease.

The research of Pishoy Gouda et al.^[27]^ indicates that the most commonly reported features and complications in LDS patients are aortic aneurysms and dissections, arterial tortuosity, high arched palate, abnormal uvula, and hypertelorism, while aneurysms of vessels other than the aorta and arterial tortuosity were some of the most frequently reported characteristics in type I LDS. Both patients in the current study had one common characteristic: they had TAAA and type B aortic dissection, and such a case has not been reported to the best of our knowledge. In addition to other artery tree aneurysms, each patient had aneurysms of the popliteal artery and hepatic artery. Also, vertebral artery tortuosity, a hallmark of type I LDS patients, was confirmed by our research (Figure 1), which was also consistent with the research results of Pishoy Gouda et al.^[27]^ As for relatively rare symptoms in type I LDS, like joint laxity, arachnodactyly, pectus deformity, early onset osteoarthritis, and osteochondritis dissecans, they were more observed in types II-V LDS patients.

In contrast to the TGFBR2 group, the average age at the onset of vascular disease was approximately 31 years in the TGFBR1 cohort^[6]^. The median survival time for LDS is 37 years, and 67% of patients died due to dissection of the thoracic aorta, 22% due to abdominal aortic dissection, and 7% due to intracranial bleeding^[5]^. In the current case, proband 1 died at 36 years of age due to the onset of acute Type B aortic dissection and failure to get any treatment. Despite suffering from TAAA, acute Standford Type B dissection, and cerebral bleeding, proband 2 recovered nicely after receiving efficient surgical therapies.

Limited guidelines have yet to be reported for managing aortic disease in individuals with LDS^[6]^. It is anticipated that early therapy will lengthen the patient's lifespan. Reportedly, arterial disorder can be treated successfully through vascular interventions with low rates of intraoperative mortality^[5]^. Aftab et al. reported that the survival rate of 33 LDS patients after the first and second year of operation (*i.e.*, open surgery) was about 90% and 80%, respectively. In the series, only six patients obtained endovascular repair: two underwent emergency TEVAR, and four underwent intervention after the first open surgery^[9]^. The work of Beaulieu et al.^[13]^ showed that treating LDS patients with consistent surveillance will be safe and effective via open and endovascular procedures. In the case of aortic diseases, the endovascular stent graft technology can be used for patients with genetic or heritable conditions, that is, when they are at high risk and have life-threatening conditions or can be used as a bridge to future definitive repair, or for the patients with a previously implanted Dacron graft^[28,29]^.

In the current investigation, the patient (proband 2) was treated for TAAA, TEVAR, and then embolism and stayed well for almost two years. Multiple surgeries are available for LDS patients^[29]^, and patients require persistent follow-ups after surgery.

### Limitations

In our study, Proband 2 (JX002-II1) underwent two operations and is currently in excellent health. But Proband 1 (JX001-II1) died unexpectedly. Two cases may not be representative, even if we successfully saved one of them. This study just only proved that endovascular operations could be a feasible option for LDS patients with aortic diseases, and further research needs more cases in the future.

## CONCLUSION

Two novel SNP variants of the TGFBR1 gene are identified in two pedigrees with clinical characteristics suggestive of LDS. Based on the study's findings, endovascular surgery can be a feasible option compared to other surgical methods, and families of LDS patients would benefit from endovascular therapy.
